# The choice of dissection or preservation of the inferior pulmonary ligament after an upper lobectomy: a systematic review and meta-analysis

**DOI:** 10.1186/s12957-019-1777-3

**Published:** 2020-01-04

**Authors:** Hao Lv, Rui Zhou, Xianghong Zhan, Dongmei Di, Yongxian Qian, Xiaoying Zhang

**Affiliations:** Department of Cardiothoracic Surgery, The third affiliated hospital of Soochow University, 185 Juqian street, Changzhou, 213000 Jiangsu Province China

**Keywords:** Inferior pulmonary ligament, Dissection, Preservation, Upper lobectomy, Meta-analysis

## Abstract

**Background:**

The necessity of the inferior pulmonary ligament (IPL) dissection after an upper lobectomy remains controversial. This meta-analysis aimed to evaluate whether this accessional procedure could reduce the postoperative complications and improve outcomes.

**Methods:**

PubMed, Embase, Ovid, Cochrane Library, CBM, and CNKI databases were searched for the relevant studies which compared the dissection with preservation of IPL during the upper lobectomy. The Review Manager 5.3 software was used for this meta-analysis.

**Results:**

Three RCTs and five CCTs were included in this meta-analysis. These studies contained a total of 610 patients, in which 315 patients received a pulmonary ligament dissection (group D) after the upper lobectomy, while the other 295 patients preserved the pulmonary ligament (group P). No significant difference was demonstrated between the group D and group *P* in terms of drainage time after surgery (MD 0.14, 95%CI − 0.05 to 0.33, *P* = 0.15), rate of postoperative dead space (OR 1.33, 95%CI 0.72 to 2.46, *P* = 0.36), rate of postoperative complications (OR 1.20, 95%CI 0.66 to 2.19, *P* = 0.56). However, the pooled comparison revealed a greater change of the right main bronchial angle (MD 5.00, 95%CI 1.68 to 8.33, *P* = 0.003) in group D compared with group P, indicated that the dissection of IPL may lead to a greater distortion of bronchus.

**Conclusions:**

This meta-analysis confirmed that the dissection of IPL do not effectively reduce the postoperative complications and improve the prognosis. Therefore, it is not necessary to dissect the IPL after an upper lobectomy.

## Background

Anatomic lobectomy with systematic lymph node dissection is considered as the standard therapy for patients with early-stage non-small cell lung cancer (NSCLC). Because of the improved long-term survival and fewer complications, the minimally invasive resection has occupied a dominant position compared with conventional thoracotomy [1]. However, when dealing with lung cancer located in different lobes, more half of the lesions are located in the upper lobes, especially in the right lobe [2, 3]. During upper lobectomy, whether the inferior pulmonary ligament (IPL) should be dissected remains a controversial issue. A traditional view has suggested that the dissection of IPL can improve the reexpansion of the inferior lobe, obliterate the free place in thoracic cavity, and then reduce the accumulation of pleural effusion [4, 5]. However, several other studies have also stated that the dissection of IPL can lead to the excessive bronchial displacement, which may be associated with the chronic dry cough or even other fatal outcomes postoperatively [6, 7].

Currently, no explicit evidence-based consensus points out the necessity of IPL dissection. Therefore, we aimed to clarify whether this unique procedure could improve the outcomes and reduce the postoperative complications through a meta-analysis of the available related clinical studies.

## Methods

This meta-analysis was conducted according to the criteria of Preferred Reporting Items for Systematic Reviews and Meta-Analyses (PRISMA) [8].

### Inclusion and exclusion criteria

Studies were included if they met the criteria as follows: (1) randomized controlled trials (RCTs), prospective or retrospective clinically controlled trials (CCTs); (2) patients received the upper lobectomy through open thoracotomy or minimally invasive approach; and (3) the comparative intervention was to dissect IPL after lobectomy. The exclusion criteria were as follows: (1) letters, editorials, case reports, and reviews; and (2) the original data could not be extracted from the articles. If multiple studies covered the overlapping data, the most recent or complete data were included.

### Search strategy

PubMed, Embase, Ovid, Cochrane Library, CBM, and CNKI databases were searched for the comparative clinical studies in Chinese or English from January 2001 to February 2019. Moreover, Google Scholar, Baidu Scholar, and reference lists of all included studies were screened for the additional articles. The following search terms were used: (“lobectomy” OR “pulmonary lobectomy” OR “pneumonectomy” OR “upper lobectomy”) AND (“pulmonary ligament” OR “inferior pulmonary ligament”) OR (“pulmonary”AND “ligament”).

### Data extraction and quality assessment

The data were independently extracted by two experienced investigators, and any conflict or disagreement arose in study selection or other related work was resolved by discussion and consensus of opinion. The following data were extracted from each study: first author, year of publication, country of origin, characteristics of patients, study design, and interventions. Primary outcomes included assessment of postoperative dead space, drainage time and volume, delayed pleural effusion, postoperative complications, and change of the main bronchial angle. In cases with missing related important data, the authors were contacted for further information via e-mails if necessary.

The Newcastle-Ottawa Quality Assessment Scale [9] was used to evaluate the quality of the CCTs. The scale consisted of three sections: patient selection, comparability between the groups, and assessment of outcomes. Each study of CCTs was assessed based on a score ranging from 0 to 9 stars. Studies with six or more stars were considered to be high-quality studies.

## Statistical analysis

Statistical analysis was performed with the Review Manager 5.3 software (Cochrane Collaboration, Oxford, UK). The odds ratio (OR) with the 95% confidence intervals (95%CI) was used for dichotomous variables, and the mean difference (MD) with its 95%CI was used for continuous variables. When the *P* value was < 0.05 and the 95% CI did not contain the value one or the value zero, the combined OR or MD was considered as statistically significant. The chi-square test was used to evaluate statistical heterogeneity among studies with significance set at *P* < 0.10, while the *I*-square (*I*^2^) test was used with significance set at *I*^2^ > 50%. If the significant heterogeneity was explored, a random effects model was applied; otherwise, a fixed effects model was applied. The sensitivity analysis was conducted to assess the stability of the pooled results by removing the low-quality studies. The high-quality CCTs with six or more stars and the RCTs were included in the sensitivity analysis. The funnel plot was used to assess the risk of publication bias.

## Results

### Search results and characteristics of included studies

**Fig. 1:**
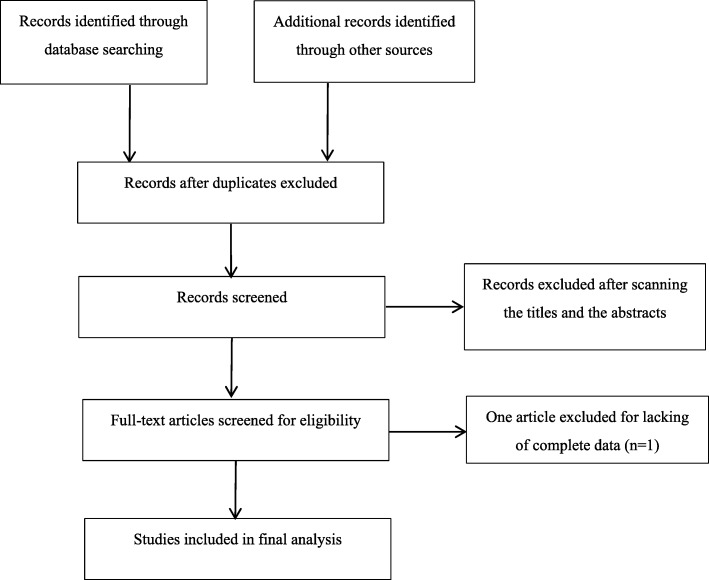
Flow diagram: literature search and selection

Figure 1 illustrates the flow chart of literature search and study screening. A total of 136 potentially relevant articles were generated through the initial search. After removing the duplicates, 124 articles were screened by scanning the titles and abstracts. Consequently, 115 irrelevant records were excluded, and the remaining nine articles were further evaluated via a full-text review. One article was excluded due to the lack of complete data. Finally, three RCTs and five CCTs that met the criteria were included in this meta-analysis. Table 1 summarizes the characteristics of these eight included studies [6, 7, 10–15]. In these studies, a total of 610 patients were included, 315 patients received a pulmonary ligament dissection (group D) after the upper lobectomy, while the other 295 patients preserved the pulmonary ligament (group P). Four studies were published in English, while the others were published in Chinese.

**Table 1:** The basic characteristic of included studies

Study	Country	Study design	Number of patients	Group		Location		NOS score (star rating)
				D	P	Left	Right	
Bu L. [6]	China	CCT	72	33	39	27	45	******
Guo H [9].	China	CCT	69	36	33	None	69	*****
Ji LL [10].	China	CCT	100	50	50	37	63	*****
Kim DH [11].	Korea	CCT	52	31	21	None	52	*******
Matsuoka, H [12].	Japan	RCT	35	17	18	12	23	_
Qi HF [13].	China	RCT	100	50	50	None	100	_
Seok Y [7].	Korea	CCT	72	43	29	34	38	******
Wang WH [14].	China	RCT	110	55	55	56	54	_

Abbreviation: *CCT* clinically controlled trial, *RCT* randomized controlled trial, *D* dissection, *P* preservation, *NOS* Newcastle-Ottawa scale

*: star

According to the Newcastle-Ottawa Quality Assessment Scale, two researchers reached a good consensus by discussing the quality of the included CCTs. Besides, two CCTs were scored with five stars, and the others achieved six or more stars, indicating a high quality. The results were also listed in Table 1.

### Drainage time after surgery

**Fig. 2:**
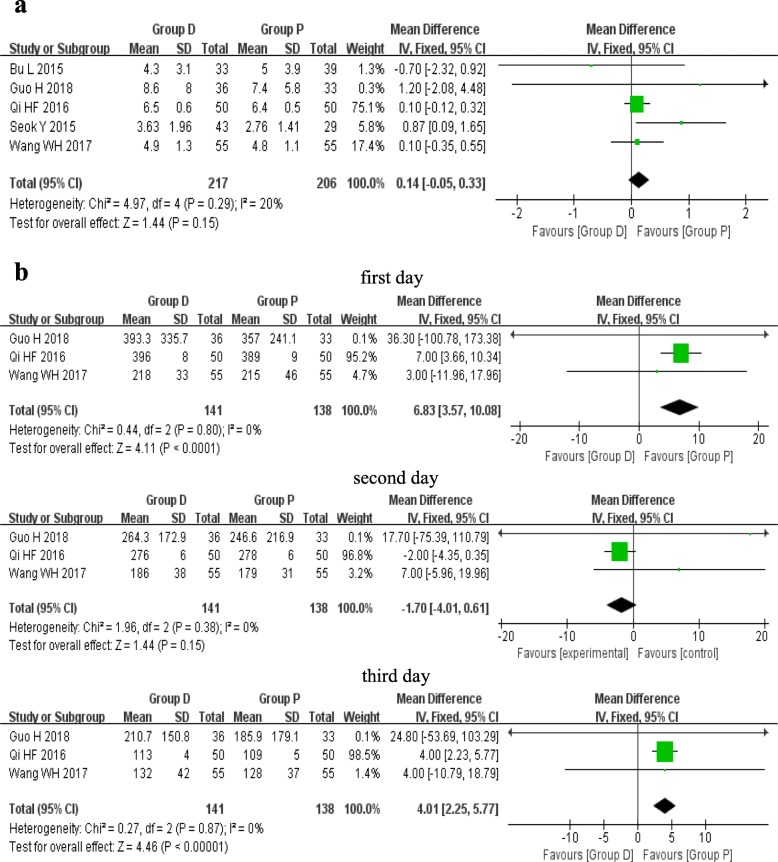
Forest plots of the whole drainage time **a** and drainage volume in the first 3 days **b** after surgery

The postoperative drainage time of chest tube was reported in five studies, including 217 patients of group D and 206 patients of group P. A fixed effects model was used because of the low heterogeneity among studies (*I*^2^ = 20%, *P* = 0.29). Data included showed no significant statistical difference between the group D and group P (MD 0.14, 95%CI − 0.05 to 0.33, *P* = 0.15) (Fig. 2).

### Drainage volume during the first 3 days after surgery

The drainage volume during the first 3 days after surgery was reported in three studies, including 141 patients of group D and 138 patients of group P. A fixed effects model was used because there was no significant heterogeneity among the three studies (*I*^2^ = 0%, *P* > 0.3). In the first day and third day, the pooled results showed a significant statistical difference between the two groups (MD 6.83, 95%CI 3.57 to 10.08, *P* < 0.0001; MD 4.01, 95%CI 2.25 to 5.77, *P* < 0.00001), suggesting an increased volume of drainage in group D compared with group P. However, data included in the second day were not sufficient to show any statistical difference between the two groups (MD − 1.70, 95%CI − 4.01 to 0.61, *P* = 0.15) (Fig. 2).

### Assessment of postoperative dead space

The assessment of postoperative dead space was reported in seven studies, which was performed with two different measurements. A direct identification of early postoperative dead space using chest X-ray tomography was reported in four studies, including 160 patients of group D and 133 patients of group P. A fixed effects model was used because there was no heterogeneity among the studies (*I*^2^ = 0%, *P* = 0.68). The pooled results showed no statistical difference between the group D and group P (OR 1.33, 95%CI 0.72 to 2.46, *P* = 0.36).

**Fig. 3:**
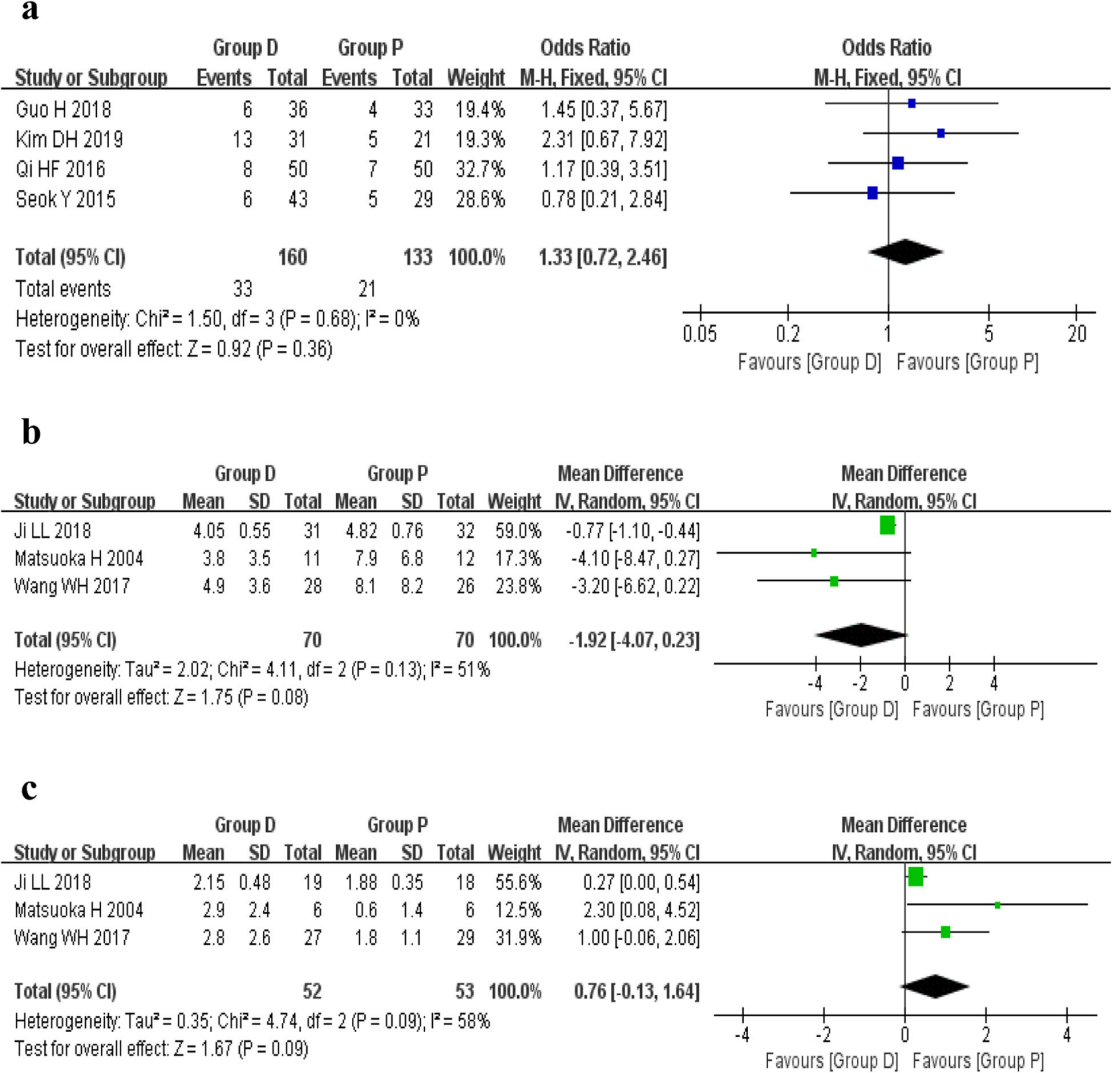
Forest plots of assessment of postoperative dead space. **a** Rate of postoperative dead space. **b** Ratio of dead space (right). **c** Ratio of dead space (left)

In other three studies, a method described by Matsuoka et al. [12] was used to evaluate the residual dead space in the left or right apex of thorax. The random effects model was used because of the high heterogeneity among the studies (*I*^2^ = 58%, *P* = 0.09; *I*^2^ = 51%, *P* = 0.13). However, neither the left side (MD 0.76, 95%CI − 0.13 to 1.64, *P* = 0.09) nor the right side (MD − 1.92, 95%CI − 4.07 to 0.23, *P* = 0.08) showed any significant statistical difference between the two groups based on the included data (Fig. 3).

### Rate of postoperative complications

**Fig. 4:**
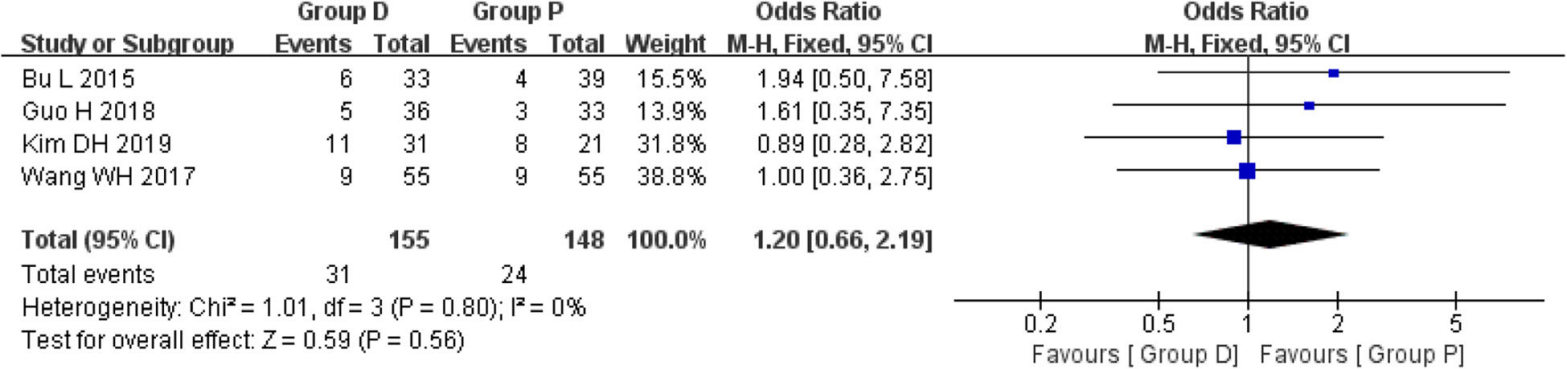
Forest plot of the rate of postoperative complications

The rate of postoperative complications was reported in four studies, including 155 patients of group D and 148 patients of group P. A fixed effects model was used because there was no significant heterogeneity among the studies (*I*^2^ = 0%, *P* = 0.80). The pooled analysis showed no statistical difference between the group D and group P (OR 1.20, 95%CI 0.66 to 2.19, *P* = 0.56) (Fig. 4).

### Change of main bronchial angle

**Fig. 5:**
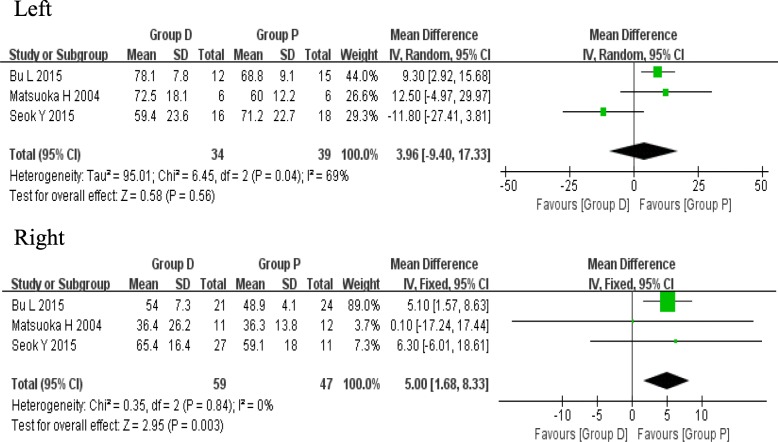
Forest plots of the change of main bronchial angle

The change of main bronchial angle postoperatively was reported in three studies, including the measurement of left and right main bronchus. For the left main bronchus, a random effects model was used (*I*^2^ = 69%, *P* = 0.04), and the pooled results showed no statistical difference between the two groups (MD 3.96, 95%CI − 9.40 to 17.33, *P* = 0.56). However, in the right comparison model, data included showed a significant statistical difference between the two groups (MD 5.00, 95%CI 1.68 to 8.33, *P* = 0.003) (Fig. 5), revealing that the dissection of IPL resulted in a greater change in the right main bronchial angle postoperatively.

### Sensitivity analysis

**Fig. 6:**
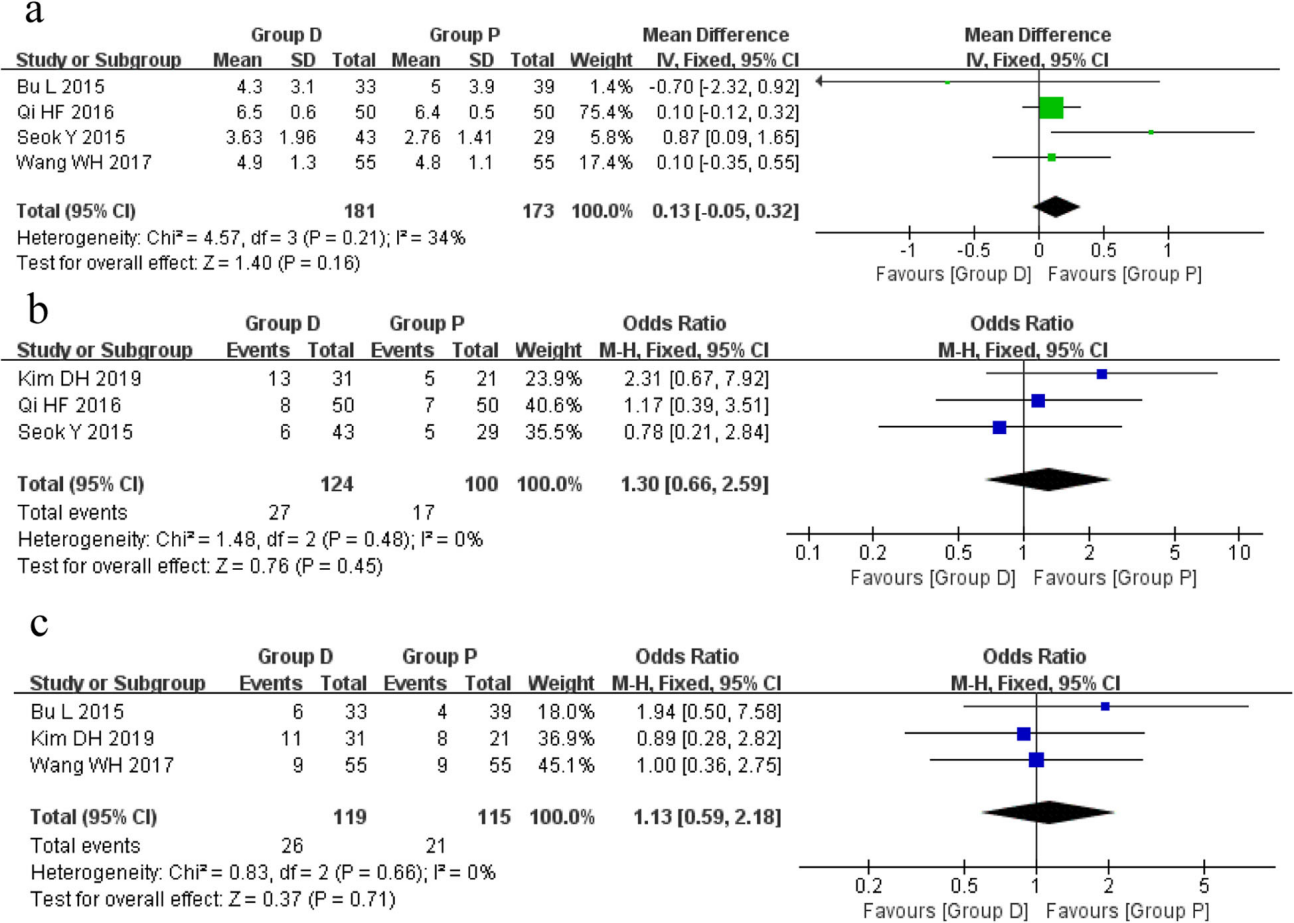
Sensitivity analysis. **a** Drainage time after surgery. **b** Rate of postoperative dead space. **c** Rate of postoperative complications

Three CCTs achieved six or more stars according to the Newcastle-Ottawa scale, and all the RCTs were included in the sensitivity analysis. The results of sensitivity analysis based on the selected studies revealed that there were no significant differences compared with those of the overall analysis (Fig. 6).

### Publication bias

**Fig. 7:**
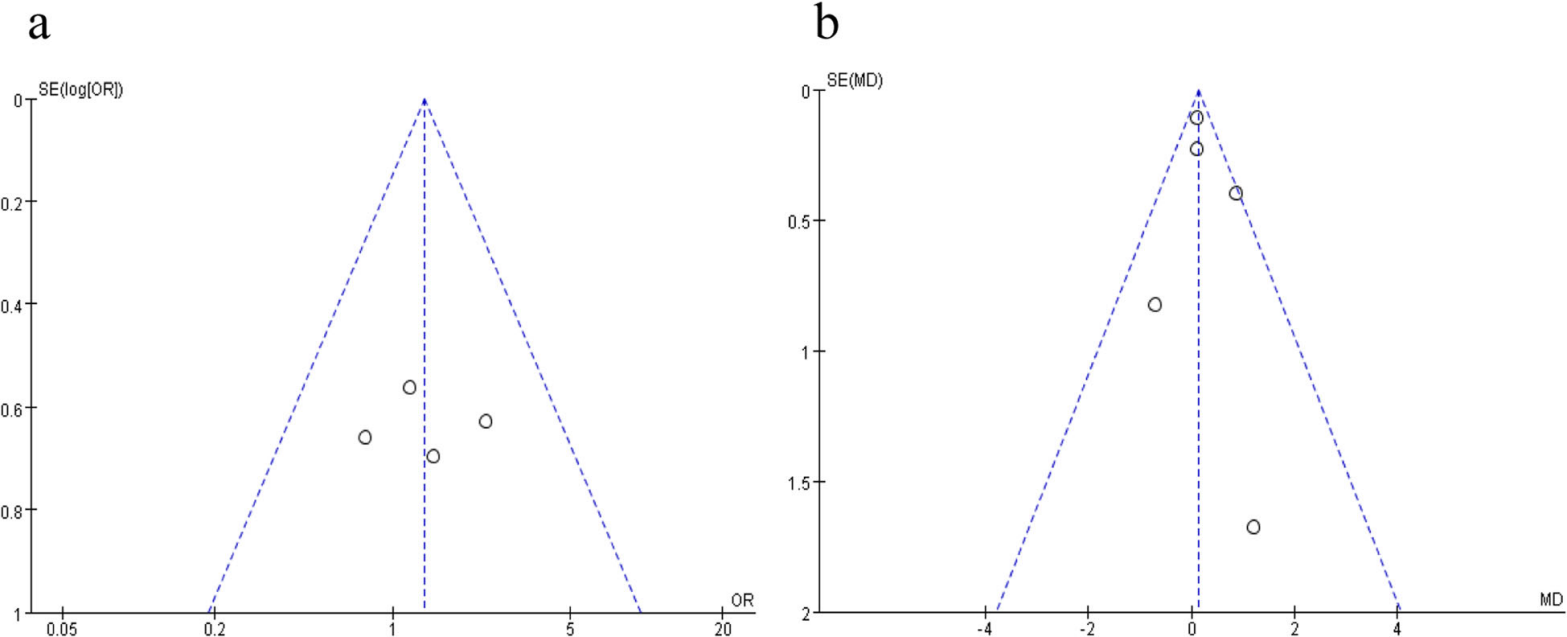
Funnel plots of the outcomes. **a** Rate of postoperative complications. **b** Drainage time after surgery

Publication bias may generate when some related studies are missing or some negative results are artificially unpublished. Figure 7 shows the funnel plots based on the outcomes. The asymmetry shown in the funnel plot based on the data of drainage time suggested the existence of potential publication bias.

## Discussion

The IPL is a double layer structure of pleura that caudally drapes from the root of lung, which plays an important role in fixing the lower lobe to the mediastinum. Dissection of IPL during upper lobectomy has been routinely performed for a long time, which is believed to be beneficial for reducing dead space and pleural effusion and then preventing empyema. However, recent studies have shown that several adverse impacts generated from this procedure, especially the tracheal deformation, have attracted more and more attention of surgeons [6, 7, 16]. Based on the abovementioned controversy, we conducted this meta-analysis and wished to get a more powerful argument in judging the necessity or effectiveness of this unique procedure.

The inevitable primary problem caused by upper lobectomy is the residual space in the thorax. The surgeons who tend to dissect the IPL seem largely based on their personal experience, which actually lacks research-based evidence. In contrast, an RCT study performed by Matsuoka et al. [12] has pointed out that the dissection of IPL makes no sense in decreasing the dead space both in right and left upper lobectomy. In our meta-analysis, the pooled comparison showed the same result as well (MD 0.76, 95%CI − 0.13 to 1.64, *P* = 0.09; MD − 1.92, 95%CI − 4.07 to 0.23, *P* = 0.08), indicating that the dissection of IPL made no apparent contribution to the obliteration of dead space.

Studies have shown that the parietal pleura covering the lower chest wall, mediastinum, and diaphragm has a stronger ability to reabsorb the fluid in the thoracic cavity [17, 18]. The division of IPL partially impairs the integrity of pleura in these areas inevitably, which may affect the reabsorption of pleural effusion. In this meta-analysis, three studies mentioned the drainage volume during the first 3 days postoperatively, and the pooled result showed that the drainage volume was significantly increased in the first and third days after the dissection of IPL (MD 6.83, 95%CI 3.57 to 10.08, *P* < 0.0001; MD 4.01, 95%CI 2.25 to 5.77, *P* < 0.00001). However, in the comparison model of drainage time after surgery, no significant difference was found between the two groups. Therefore, we believed that the dissection of IPL might increase the drainage volume in the early period postoperatively. Nevertheless, the whole drainage time was not obviously prolonged, which might be also affected by other factors, such as different numbers, diameters of chest tubes, and different volume thresholds for chest tube removal used among studies. A questionnaire survey conducted by Usuda et al. [4] in Japan has revealed that nearly 28% directors in the Department of Thoracic Surgery tend to attribute the pooling of pleural effusion to the preservation of IPL. However, a recent study performed by Kim et al. [11] has stated that no significant difference is found between the preservation and dissection groups in terms of delayed pleural effusion.

It is well known that the upward movement of residual lobes will pull the bronchial lumen after the upper lobectomy, leading to the change of bronchial angle. Consequently, the deformed bronchus may become kinked, stenotic, and even obstructed. Usuda et al. [19] have previously reported a patient who suffers from a severe bronchial stenosis after the left upper lobectomy in combination with the IPL dissection. BU et al. [6] have also found that the change in main bronchial angle after the IPL dissection is significantly greater in the left lung compared with the right lung, which may lead to the reduced pulmonary capacity and ventilation dysfunction. In addition, Seok et al. have reported a significant change between the right intermedius and middle lobe bronchus using the three-dimensional reconstruction images [7]. Unexpectedly, in our meta-analysis, the pooled comparison consisting of three studies only revealed a more significant change in the right main bronchial angle (MD 5.00, 95%CI 1.68 to 8.33, *P* = 0.003) rather than the left one. Besides the degree of measurement accuracy, we speculated that this discrepancy could also be attributed to other factors, such as the development of pulmonary fissure, the extent of hilar dissection and mobilization, which might have a greater impact on the movement of bronchus. Actually, despite the changes in bronchial angle have been discovered by these studies, the direct evidence is still missing to point out whether these changes can increase the rate or severity of abovementioned postoperative complications. In other words, it is just the speculation of researchers so far, which is not supported by any solid evidence. Therefore, more rigorous and precise studies should be carried out to provide a deeper insight into this phenomenon.

Previously, Khanbhai et al. [20] have reviewed the relevant articles in major databases and found no convincing evidence to support whether the dissection of IPL can reduce postoperative complications. In our comparison model, the postoperative complications included atelectasis, arrhythmia, pulmonary infection, empyema, and the delayed air leak. However, no significant difference was found in terms of complication rate (OR 1.20, 95%CI 0.66 to 2.19, *P* = 0.56), which seemed to be consistent with the result from Bu et al. [6]. However, the shortness in our study was that we only focused on the overall rate of complications and neglected the fact that different complications may derive from various reasons.

Undoubtedly, the reduction of pulmonary parenchyma during the lobectomy inevitably leads to the corresponding loss of pulmonary capacity and function, which may impair the postoperative recovery and prognosis [21]. However, the accessional impact on the postoperative pulmonary function caused by the IPL dissection is still uncertain. Bu et al. [6] have previously reported that the forced expiratory volume in one second (FEV1) is significantly greater in group P compared with group D at 3 months postoperatively. Meanwhile, they have found that the residual lung volume is also significantly higher in group P at 6 months, which is measured by CT scans. They have speculated that this phenomenon is associated with the enlarged angle and distortion of bronchus resulted from the IPL dissection. Similarly, results from Kim et al. [11] have shown that the IPL dissection can lead to the loss of forced vital capacity, which may cause atelectasis or dead space rather than the reexpansion postoperatively. Unfortunately, the relevant comparison was not performed in the present meta-analysis because of the insufficient data and different measurements derived from the included studies.

The anatomical location of IPL is closely related to the structures, such as descending aorta, esophagus, inferior vena cava, and inferior pulmonary vein. The potential damage to these structures during the IPL dissection must be worthy of consideration. Moreover, data from the previous studies [22, 23] have shown that several lymphatic vessels derived from the lung connected with the thoracic duct are located within the IPL, indicating the possibility of postoperative chylothorax resulted from the IPL dissection. Collectively, these abovementioned potential risks should not be simply ignored.

However, a vital issue that we could not neglect is the relationship between inferior mediastinal lymph node dissection and division of IPL during an upper lobectomy, which remains a controversial topic. Since a systematic dissection of mediastinal lymph node has been recommended as the standard procedure after lobectomy for resectable lung cancer [1, 3], the IPL is always divided when dealing with the inferior mediastinal lymph nodes, especially the obviously enlarged nodes lying on IPL or beside the esophagus. Nowadays, with the widespread use of the low-dose spiral computed tomography, more and more early-stage lung cancers are encountered in routine medical examination [24]. Accordingly, many investigators have paid attention to the nodal spread patterns and claimed that the selective lymph node dissection based on tumor location shows similar survival outcome compared with the systematic dissection in early-stage lung cancer [25–27]. Moreover, it can also reduce injury and shorten the operation time, which is the trend of precision medicine. Unfortunately, the included studies in our meta-analysis did not clearly indicate whether patients in group P all received the selective lymph node dissection without dividing IPL. Actually, a few patients in group P ultimately reached pathological stage III or IV after surgery, indicating that there was an incomplete lymph node dissection.

Undeniably, there are some limitations in our meta-analysis. First, the present study only included three RCTs, and almost all the included trails had a small sample size, which might generate a low statistical power. Second, only full-text articles in English or Chinese language were included, and the bias existed when literatures published in other languages were not identified. Thirdly, all the included studies were from Asia, and the representativeness of the results might be regionally. Fourth, different operation techniques and preoperative management strategies were used among different studies, which inevitably led to the heterogeneity. In consideration of the limitation of sample size, we did not conduct the subgroup analysis between the thoracoscopy and thoracotomy. In future, more large-scale precise RCTs from more areas are needed to confirm these findings.

## Conclusions

Our results indicated that the IPL dissection after an upper lobectomy did not effectively reduce the postoperative complications and improve the prognosis. Meanwhile, considering the fact that the remnant bronchus experiences a greater deformation after the IPL division, prospective research is essential to confirm the further effect derived from this alteration.

## Data Availability

All the data analyzed in this study are obtained from the original articles.

## Abbreviations

CCT: Clinically controlled trial
D: Dissection
FEV1: Forced expiratory volume in one second
IPL: Inferior pulmonary ligament
NOS: Newcastle-Ottawa scale
NSCLC: Non-small cell lung cancer
P: Preservation
PRISMA: Preferred Reporting Items for Systematic Reviews and Meta-Analyses
RCT: Randomized controlled trial
UK: United Kingdom
